# Influence of landscape characteristics and submerged aquatic vegetation on sediment carbon and nitrogen storage in shallow brackish water habitats

**DOI:** 10.1038/s41598-025-92217-z

**Published:** 2025-03-06

**Authors:** Sofia A. Wikström, Betty Gubri, Maria E. Asplund, Martin Dahl, Martin Gullström, Joakim P. Hansen, Linda Kumblad, Emil Rydin, Andrius Garbaras, Mats Björk

**Affiliations:** 1 Stockholm University Baltic Sea Centre, 106 91 Stockholm, Sweden; 2 Department of Ecology, Environment and Plant Science, Stockholm University, 106 91 Stockholm, Sweden; 3 Department of Biological and Environmental Sciences, University of Gothenburg, Kristineberg, Fiskebäckskil, Sweden; 4 School of Natural Sciences, Technology and Environmental Studies, Södertörn University, 141 89 Huddinge, Sweden; 5 Isotope Research Laboratory, Centre for Physical Sciences and Technology, Savanoriu 231, 02300 Vilnius, Lithuania

**Keywords:** Blue carbon, Shallow bays, Coastal lagoons, Macrophytes, SAV, Element cycles, Marine biology

## Abstract

While marine seagrass habitats are acknowledged as sinks for carbon and nutrients, much less is known about sequestration in brackish-water vegetation. Here, we quantify the amount of organic carbon (C_org_) and total nitrogen (TN) in shallow bay sediments (0–25 cm) in the brackish Baltic Sea and assess how it varies with morphometric isolation from the sea, catchment characteristics and abundance of brackish-water vegetation. The sedimentary C_org_ and TN content per surface area varied across the bay isolation gradient (mean C_org_: 2500–4600 g/m^2^; mean TN: 320–570 g/m^2^), with enclosed bays having the highest percentage content of C_org_ and TN, but low sediment density (< 0.1 g cm^3^), while open bays had more compact sediment with lower percentage content of C_org_ and TN. The influence of catchment and vegetation characteristics on the sediment C_org_ and TN content was less clear, suggesting that coastal morphology affecting hydrodynamic exposure is an important determinant of C and TN accumulation in brackish-water bays. The results show that morphometrically isolated shallow coastal areas constitute significant sinks for carbon and nitrogen, which should be considered in management and in any regional estimates of blue carbon and nutrient sequestration functions.

**Supplementary Information:**

The online version contains supplementary material available at 10.1038/s41598-025-92217-z.

## Introduction

**Fig. 1:**
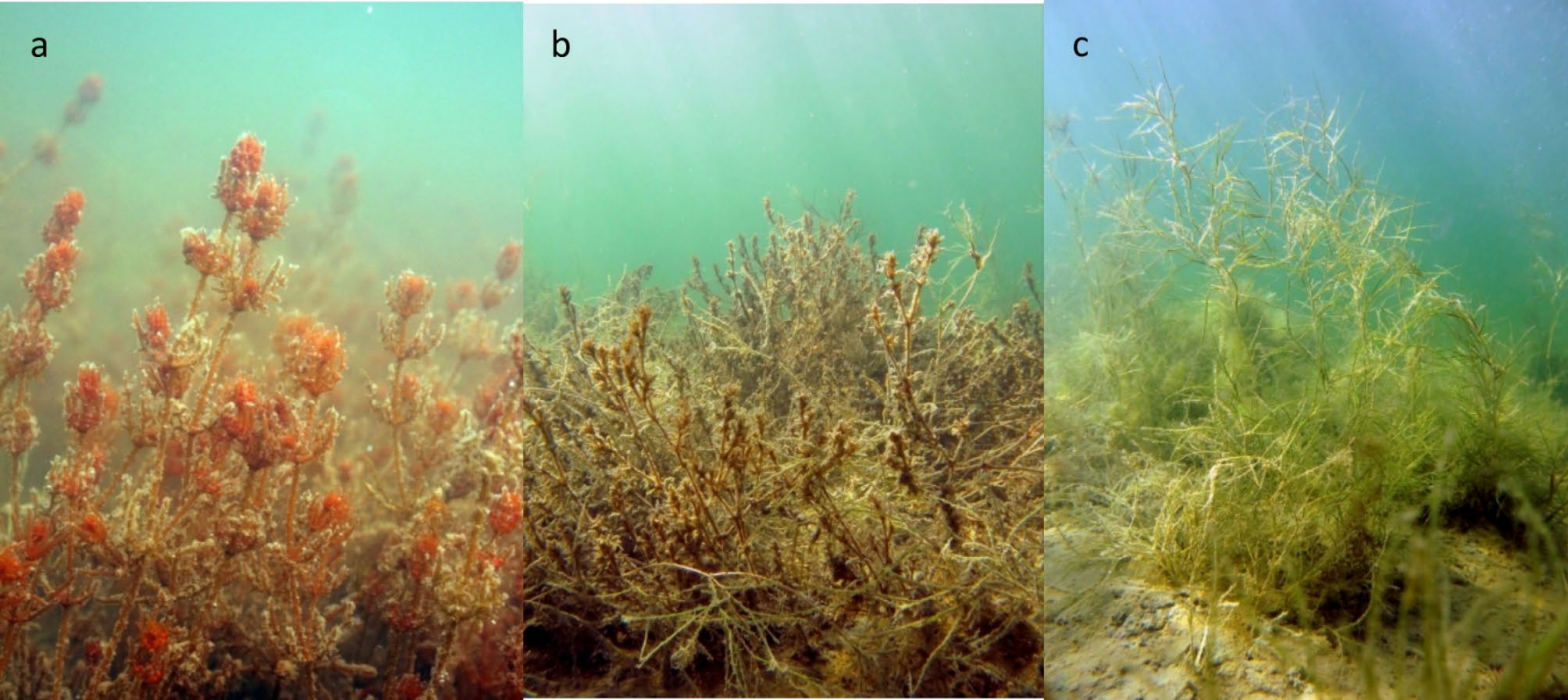
Examples of estuarine vegetation; *Chara tomentosa* (**a**), *Najas marina* (**b**), and *Stuckenia pectinata* (**c**). Photos: Joakim P. Hansen

Seagrass meadows are recognised as significant sinks for carbon and nutrients. High accumulation rates of organic matter and slow decomposition result in large carbon stocks in many seagrass meadows^1–3^. This is why protection and restoration of seagrass meadows are increasingly regarded as a measure for climate change mitigation^4^. Seagrass meadows also enhance nitrogen removal processes in coastal areas, improving local water quality and functioning as a filter for incoming nutrients from surrounding catchment areas^5,6^. Compared to seagrass systems, much less is known about carbon and nutrient sequestration in other underwater vegetation habitats. Seagrass constitutes the dominating group of plants in marine ecosystems, but in estuaries and other areas with brackish water they are partly replaced by a diverse group of plants and algae originating mainly from freshwater (Fig. 1). This brackish-water vegetation can cover large areas in the low-saline parts of coastal lagoons, estuaries, river deltas and inland seas^7–9^, but few studies have quantified carbon or nutrient stocks in such habitats and they are often not accounted for in regional estimates of coastal blue carbon sequestration^10,11^. Many brackish-water systems are heavily affected by nutrient pollution and sediment from their watersheds and climate-driven sea level change, with resulting losses of submerged vegetation^12,13^. A better understanding of the capacity of brackish-water vegetation habitats as carbon and nutrient sinks will improve regional and global estimates of coastal blue carbon and nutrient sequestration functions in coastal areas and can support management, conservation and restoration of coastal ecosystem functions and services.

In seagrass habitats, sedimentary carbon stocks vary strongly between sites, depending on factors regulating production, sedimentation and remineralisation of organic matter^14^. For instance, the geomorphology of the coastal landscape determines the degree of exposure to hydrodynamic forces such as waves, tides and currents, affecting patterns of sedimentation and erosion. Dense and extensive seagrass meadows in areas characterised by low hydrodynamic energy can be accumulation hotspots, resulting in organic-rich sediments and large deposits of allochthonous organic carbon and nutrients^14–17^. The species composition and canopy characteristics of the seagrass communities can also have a significant impact on the accumulation and storage of carbon in seagrass habitats^14,18,19^. Seagrass vegetation reduces hydrodynamic energy, enhances trapping and accumulation, and reduces erosion of organic matter, and several species produce large root-rhizome systems of highly recalcitrant organic matter that accumulate in the sediment. Similar to seagrass, submerged brackish and freshwater vegetation have been shown to trap sediment and reduce sediment resuspension^20^, but few studies have evaluated what this means for carbon and nutrient sequestration in these habitats.

The Baltic Sea is one of the largest brackish-water areas in the world, where the occurring seagrass species, mainly *Zostera marina*, is largely confined to moderately wave-exposed areas, forming meadows with low sediment organic carbon content^21–23^. More wave-sheltered vegetation habitats have long been overlooked as potential sinks for carbon and nutrients in the region, but a recent study showed that shallow bays of the Baltic Sea sequester significant amounts of carbon and nutrients, with sediment stocks and accumulation rates of organic carbon, nitrogen and phosphorus comparable to those in seagrass habitats in adjacent marine coastal areas^48^. Beyond that study, very little is known on carbon and nutrient storage in sheltered brackish-water vegetation in the Baltic Sea, in particular how it varies in the coastal seascape. Most of the coastline in the Baltic Sea is characterised by postglacial land uplift, shoreline displacement, and a complex seascape of sheltered areas and bays that are gradually isolated from the sea^24^. This creates a natural succession of bays affecting water exchange with the sea, wave exposure, water temperature and ecological functions of the bay systems that can be predicted to affect cycling and accumulation of carbon and nutrients in several different ways. For instance, the formation of a threshold at the mouth of the bay changes the deposition environment and likely increases the sedimentation rate of allochthonous organic matter of both terrestrial and marine origin. Subsequent changes in vegetation composition can change the local production of organic carbon and the function of the estuarine vegetation in enhancing carbon accumulation in the sediment. This means that the potential for carbon and nitrogen sequestration is likely to vary across the coastal seascape of bays in various successional stages, creating a good study system to evaluate how the potential for carbon and nutrient storage varies in brackish-water seascapes.

**Fig. 2:**
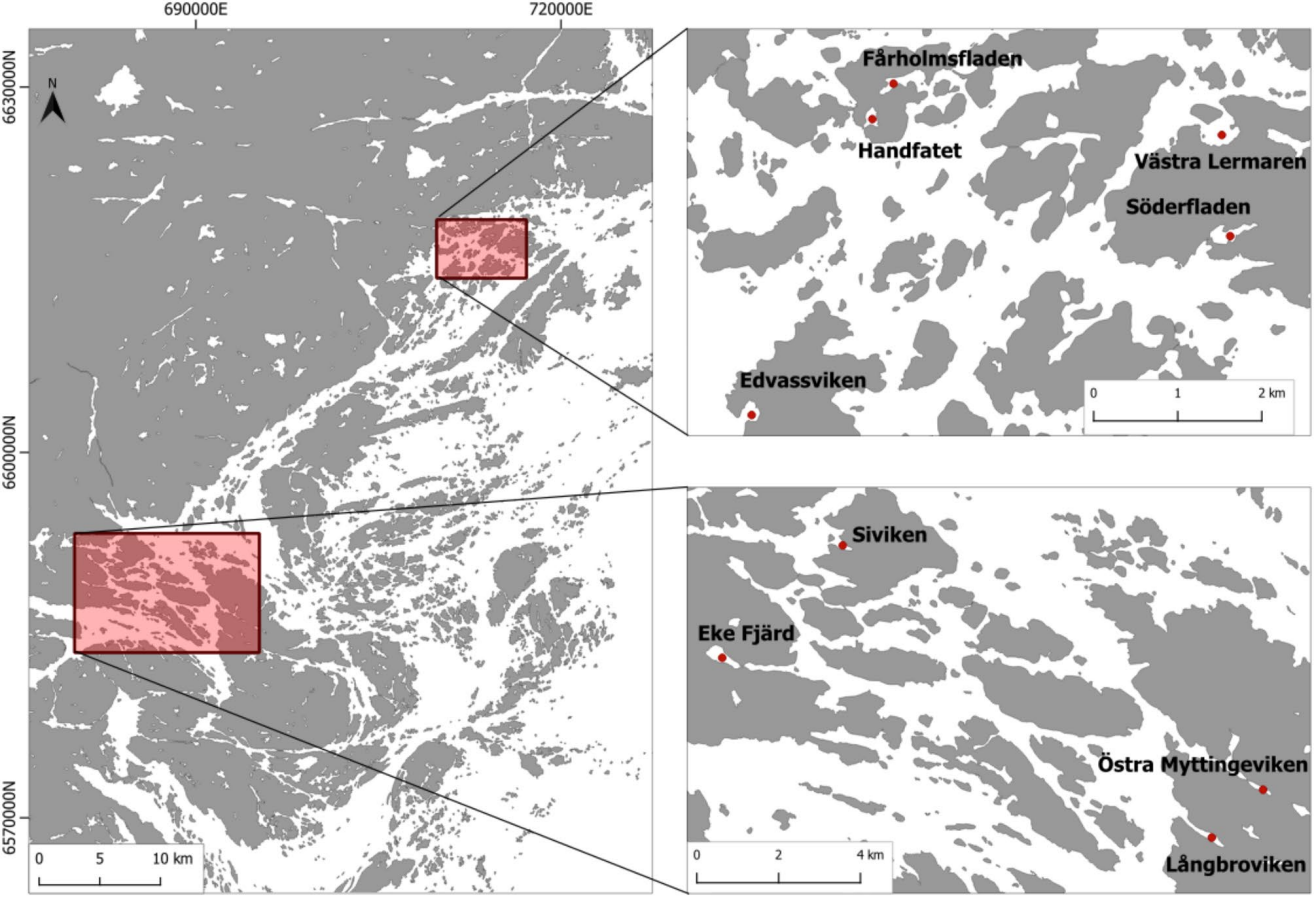
Map of the study areas and the nine investigated semi-enclosed bays located in the Stockholm archipelago, Baltic Sea. The southern area is close to the outflow of the large lake Mälaren and has lower and more variable salinity than the northern area. Map created with QGIS (v3.34.3), with background layer from OpenMapTiles (https://openmaptiles.org/)

Here, we study how the sediment content and stocks of carbon and nitrogen vary in a gradient of topographic openness in shallow semi-enclosed Baltic Sea bays (Fig. 2). Topographic openness (calculated from the cross-section area of the bay opening and the bay area) has been shown to be a good predictor of water retention time of the bays^25^. It is a useful morphometric character to capture the strong gradient in wave impact created by the gradient in isolation from the sea, since low topographic openness implies a shallow and narrow entrance that decrease the hydrodynamic energy of waves and currents entering into the bay. We further assess the relationship between sediment carbon and nitrogen and a number of indicators of the surrounding landscape, and the characteristics of the submerged vegetation. Finally, we test for differences in C and N content in the surface sediment between patches with and without submerged vegetation within bays.

## Results

### Landscape variables and underwater vegetation

The topographic openness of the bays varied from < 0.01 to 0.59 (Table 1). This corresponds to a gradient from enclosed bays with very shallow entrances, almost completely closed by reed, with water retention time of about 26 days, to semi-enclosed bays with shallow but open entrances with water retention time of about one day. The bays were shallow (mean depth of 1.5–2.4 m) and small (0.04–0.19 km^2^), with local catchment areas varying from 0.1 to 6.4 km^2^ (Table 1). Coniferous forest was the dominating land cover in all catchments, covering between 50 and 90% of the catchment area, while the remaining land cover being open or arable land and residential buildings.

The submerged aquatic vegetation (SAV) consisted of a mixture of charophytes (*Chara aspera*,* C. baltica*, *C. globularis*, *C. tomentosa* and *C. virgata*), vascular plants (*Ceratophyllum demersum*, *Callitriche hermaphroditica*, *Myriophyllum spicatum*, *Najas marina*, *Potamogeton perfoliatus*, *P. pusillus*, *Ranunculus circinatus*, *Stuckenia pectinata* and *Zannichellia palustris*), one bryophyte (*Fontinalis antipyretica*) in one bay, and some occurrences of the seaweed *Fucus vesiculosus* in two of the bays. The species composition differed between the bays, with extensive stands of *Najas marina* and/or charophytes dominating in most of the enclosed bays (except Eke fjärd) and larger dominance of tall-growing species such as *Myriophyllum spicatum*, *Potamogeton perfoliatus* and/or *Stuckenia pectinata* in the three most open bays (Table 1). The mean vegetation cover was intermediate (40–50%) or high (> 70%), except in Eke fjärd with low mean cover (< 20%; Table 1). In addition to the submerged vegetation, all bays were fringed by reed beds covering between 3 and 29% of the bay area.

### Sediment characteristics and Corg and TN content

**Fig. 3:**
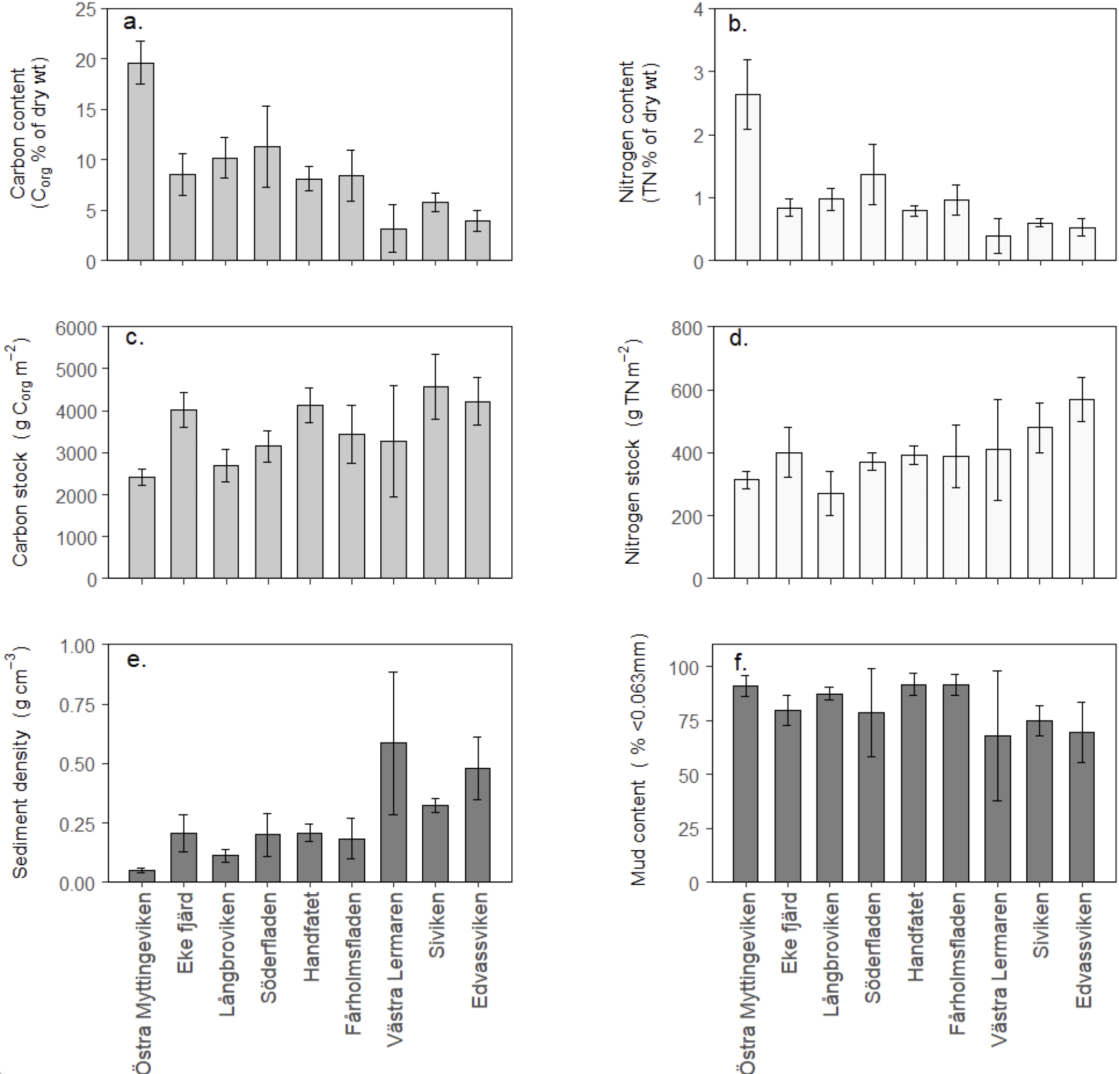
Sediment stocks and % content of C_org_ and TN, sediment density and % content of mud (< 0.063 mm) in the upper 25 cm of sediment in the nine studied bays, ordered from smallest to largest bay topographic openness (Ea). Data are means and standard deviation for 3–4 cores in each bay

The mean % C_org_ (of dry weight) differed significantly between bays, from around 3% to as high as 20% C_org_ (F_8,26_ = 19.19, *p* < 0.001; Fig. 3a), and the mean % TN showed a similarly large range (F_8,26_ = 12.34, *p* < 0.001, Fig. 3b). The highest % C_org_ and TN were recorded in bays with low topographic openness, which were characterised by sediment with very low sediment density (< 0.1 g/cm^3^; Fig. 3e) and high mud content (< 0.063 mm; Fig. 3f). In these bays, the surface sediment consisted of a fluffy layer dominated by loose, decomposing organic matter and the very high C_org_ and TN content recorded in the most enclosed bay (Östra Myttingeviken) resulted from the fluffy layer extending down to the deepest part of the cores (12.5–25 cm; Supplementary Fig. 1).

The mean C_org_ and TN stocks in the upper 25 cm also differed significantly between the bays (F_8,26_ = 4.885, *p* < 0.001 for C_org_; F_8,26_ = 4.392, *p* = 0.002 for TN), but the differences were smaller and showed the opposite pattern to % C_org_ and TN content (Fig. 3c and d). Mean C_org_ stocks ranged between 2400 and 4600 g C_org_ m^− 2^ and mean nitrogen stocks between 270 and 570 g TN m^− 2^, with the lowest densities in some of the most enclosed bays.

**Table 1:** Landscape and vegetation characteristics of the nine studied bays

Bay	Openness (Ea)	Bay area (km^2^)	Catchment area (km^2^)	SAV cover (%)	Dominating SAV species^a^ (S = number of species)
Östra Myttingeviken	0.003	0.06	3.02	75	*Najas marina* (S = 5)
Eke fjärd	0.009	0.19	6.54	17	*Myriophyllum spicatum*, *Ceratophyllum demersum* (S = 4)
Långbroviken	0.013	0.10	2.75	46	*Najas marina* (S = 7)
Söderfladen	0.017	0.07	0.91	80	*Chara* spp., *Najas marina* (S = 5)
Handfatet	0.056	0.03	0.11	88	*Chara* spp., *Najas marina* (S = 10)
Fårholmsfladen	0.101	0.07	0.21	78	*Chara* spp., *Najas marina* (S = 7)
Västra Lermaren	0.259	0.13	0.51	43	*Callitriche hermaphroditica*, *Myriophyllum spicatum*, *Fucus vesiculosus* (S = 7)
Siviken	0.403	0.08	0.64	42	*Najas marina*, *Potamogeton perfoliatus*, *Stuckenia pectinata* (S = 6)
Edvassviken	0.591	0.04	0.70	43^b^	*Myriophyllum spicatum*, *Potamogeton perfoliatus* ^b^

Topographic openness (Ea) was calculated from the cross-section area of the bay opening and the bay surface area (see Methods). Cover, dominating species and total number of species of submerged aquatic vegetation (SAV) were taken from vegetation surveys of the entire bay, except for in Edvassviken where the vegetation was only surveyed around the sediment sampling stations

^a^Species together constituting > 75% of the total vegetation cover in the bay

^b^Based on the vegetation survey at the four sediment sampling stations

### Relating landscape variables, vegetation and sediment characteristics to sediment C_org_ and TN

A principal component analysis (PCA) bi-plot assessing the relationship between landscape, vegetation and sediment characteristics and the nine study sites showed that the sediment variables (sediment dry bulk density and mud content) were correlated with bay openness and that all measures of catchment area (total area and area of forest, open land and farmland) were mutually correlated (Supplementary Fig. 2). The different variables describing the submerged vegetation (cover and biomass of submerged vegetation and filamentous algae in the entire bay and at the sampling stations) were mutually correlated. Partial least squares (PLS) regression modelling resulted in significant one-component models for % C_org_ content, % TN content and TN stocks, while C_org_ stocks did not show any relationship to the predictor variables. The cross-validated variance (Q^2^ statistics) was 50.3% for C_org_ content, 19.1% for TN content and 25.5% for TN stocks. The Q^2^ values hence showed predictability, as they were higher than the significant limit level of 5%. The cumulative fractions of all predictor metrics combined (R^2^_y_ cum) explained a moderate to high part of the variation (i.e. 70.8% for C_org_ content, 59.5% for TN content and 60.6% for TN stocks), which shows that the significant models displayed a relatively high degree of determination.

**Fig. 4:**
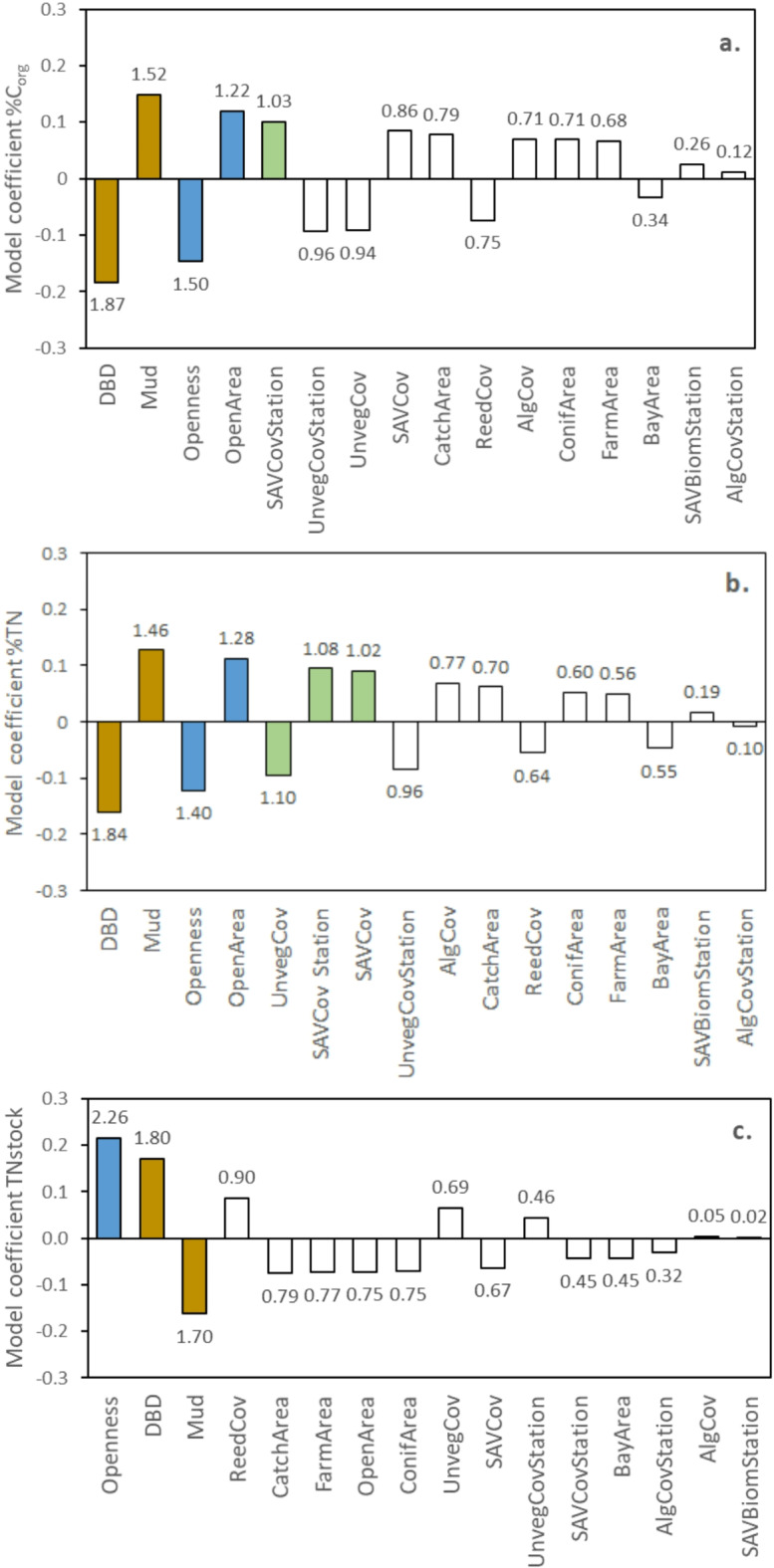
Coefficient plots of partial least squares (PLS) regression models of % C_org_ content (**a**), % TN content (**b**) and TN stocks per area (**c**) in the upper 25 cm of sediment. Predictor variables are ranked in order from most (to the left) to least influential (to the right). Note that the vegetation variables represent two different scales; the entire bay and around the sediment sampling stations (“…Station”). Numbers above or below bars show VIP (variable influence on the projection). Blue (landscape predictors), green (vegetation predictors) and brown (sediment predictors) bars represent independent variables with VIP values above 1, meaning they contribute significantly (above average) to the model of a certain response variable. White bars represent predictors contributing less than average to the overall model performance for the response variable. Abbreviations of predictor names are as follows: *Openness* Bay topographic openness (Ea), *BayArea* Area of bay, *CatchArea* Catchment area, *ConifArea* Area of coniferous forest in catchment, *OpenArea* Area of open land in catchment, *FarmArea* Area of farmland in catchment, *SAVCov* Cover of submerged aquatic vegetation, *ReedCov* Cover of aquatic reed, *AlgCov* Cover of filamentous algae, *UnvegCov* Cover of bare sediment, *SAVCovStation* Cover of submerged aquatic vegetation at sampling stations, *AlgCovStation* Cover of filamentous algae at sampling stations, *UnvegCovStation* Cover of bare sediment at sampling stations, *SAVBiomStation* Above-ground biomass of submerged aquatic vegetation at sampling stations, *DBD* Sediment dry bulk density, *Mud* Mud content in sediment. See Methods for more details on the predictor variables.

Sediment properties in terms of sediment density (negatively related) and mud content (positively related) showed the strongest contributions to both the C_org_ and TN content models (Fig. 4a and b). Landscape metrics were also important for the C_org_ and TN content models; bay openness (negatively related) and the area of open land in the bay catchment (positively related) contributed significantly to model performance. In addition, cover of vegetation at the sediment sampling stations (positively related) contributed to both the C_org_ and TN content model and the vegetation cover and cover of bare sediment in the entire bay (the first positively and the second negatively related) contributed to the TN content model.

Corresponding to the C_org_ and TN content models, bay openness and the two sediment metrics were the strongest predictors for the TN stocks model, but their respective influence was in an opposite direction (Fig. 4c). Bay openness and sediment dry bulk density were positively related to the TN stocks, whereas mud content was negatively related to the TN stocks.

### Small-scale variation between patches with and without vegetation

**Fig. 5:**
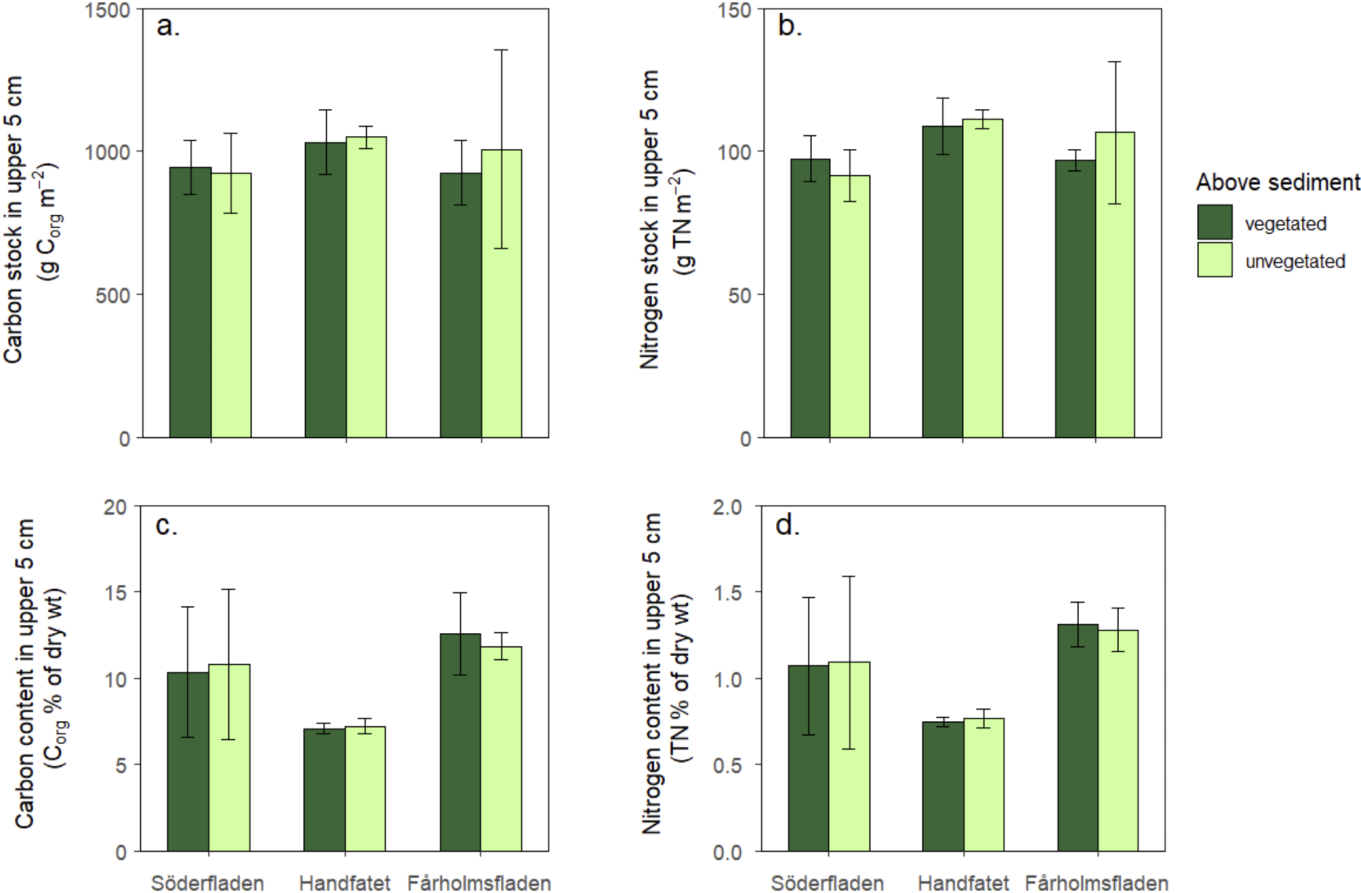
Sediment stocks (**a**, **b**) and % content (**c**, **d**) of C_org_ and TN in the upper 5 cm of the sediment in patches with and without submerged vegetation (*Najas marina* or *Chara tomentosa*) in three bays. Data are means and standard deviations for four cores in each type of patch in each bay.

There were no significant differences in C_org_ or TN stocks or % C_org_ or TN content in the surface sediment (upper 5 cm) between patches with and without submerged vegetation (Table 2; Fig. 5). The vegetation around the sampling points consisted of *Chara tomentosa* in Söderfladen (mean aboveground biomass: 243 g dry weight per m^2^), *Najas marina* in Handfatet (104 g dw per m^2^) and *Najas marina* with small amounts of *Chara tomentosa* in Fårholmsfladen (81 g dw per m^2^).

**Table 2:** Results from two-way ANOVAs testing the effect of patch type (vegetation or no vegetation) and Bay on sediment stocks and % content of C_org_ and TN in the upper 5 cm of the sediment

	C_org_ stocks^a^				TN stocks^a^		
	df	MS	F	p	MS	F	p
Vegetation	1	4.1 × 10^3^	0.140	0.713	26.7	0.182	0.675
Bay	2	2.4 × 10^4^	0.808	0.461	486.4	3.316	0.059
Veg x Bay	2	5.5 × 10^3^	0.187	0.831	118.1	0.805	0.463
Residuals	18	2.9 × 10^4^			146.7		

	% C_org_ content				% TN content		
	df	MS	F	p	MS	F	P
Vegetation	1	8.2	0.345	0.564	0.04	0.001	0.970
Bay	2	357.1	15.072	< 0.001	307.12	10.453	< 0.001
Veg x Bay	2	0.3	0.012	0.988	2.17	0.074	0.929
Residuals	18	23.7			29.38		

The analyses of % C_org_ and TN were done on rank-transformed data due to widely different variances between the bays

^a^The analysis includes one outlier. When removing the outlier, there was a significant effect of bay (C_org_ stock: F_2,17_ = 3.729, *p* = 0.045; TN stock: F_2,17_ = 8.509, *p* = 0.003), but still no effect of vegetation or significant interaction

### Sedimentary δ^13^C and C/N ratio

The mean sedimentary δ^13^C in the upper 25 cm layer was between − 26 and − 20 and the mean C/N ratio between 7.4 and 10.6 in the studied bays (Supplementary Fig. 3). Previous studies from shallow bays in the Baltic Sea have shown that overlapping δ^13^C for terrestrial vegetation (− 29 to − 22^27^) and Baltic Sea coastal phytoplankton communities (− 24 to − 22^27,28^) makes it difficult to apply formal mixing models to estimate the relative contribution of autochthonous and different allochthonous sources for organic matter deposited in benthic vegetation^27^. However, the fact that the sedimentary δ^13^C in the bays were closer to the signal from these sources than the considerably higher values recorded for submerged aquatic vegetation in Baltic Sea coastal bays (− 15 to − 8) suggests that a lot of the sedimentary carbon is derived from land or from pelagic production, with relatively small addition of organic matter from the local vegetation communities. The low sediment C/N ratios point to a large contribution of phytoplankton to the sediment organic matter in the bays, since phytoplankton are more enriched in N relative to C compared to terrestrial plants and aquatic vegetation^29^ and typically display C/N ratios between 5 and 10.

## Discussion

Our results indicate that coastal morphology (here measured as topographic openness), which affects local hydrodynamic conditions such as waves and currents, has a large impact on sediment content (as percentage of dry weight) of C_org_ and TN in shallow vegetated bay habitats of the Baltic Sea. The large influence of hydrodynamic exposure is coherent with what has been observed in coastal habitats without vegetation, or those dominated by small and opportunistic seagrass species^16^. In addition, the relationships between mud content, sediment density and C_org_ content in the studied bays was well in line with what is typically seen in marine sediments, such as those in the open Baltic Sea^30^.

While C_org_ and TN content increased markedly with increasing isolation of bays from the sea (i.e. decreasing topographic openness), the sediment TN stocks in the upper 25 cm showed the opposite pattern and the C_org_ stock was not significantly related to bay openness according to the PLS analysis. This contrasts with several studies from seagrass habitats, which show that C_org_ and TN stocks increased with decreasing current velocity or wave exposure^14,16,17,31^. A likely explanation for the difference is that our study focused on habitats with low or very low hydrodynamic exposure, where the very low sediment density in the most enclosed bays resulted in low content (per sediment volume) of C_org_ and TN and hence similar C_org_ stocks and lower TN stocks compared to the more open bays. Similar patterns of decreasing or constant C_org_ and TN stocks with decreasing wave exposure have been documented from sheltered to semi-sheltered *Zostera marina* habitats^15,17^, while studies showing increasing C_org_ stocks with increasing current velocity or wave exposure have focused on sandy seagrass sediments with C_org_ from around 0 to below 3%^16,31^. This means that while C_org_ and nutrient content (in % of sediment weight) in vegetated coastal sediments can be expected to increase linearly with decreasing hydrodynamic impact, a very high %C_org_ content in the sediment does not always translate to correspondingly high stocks if the sediment density is low.

It is important to note that we report mean C_org_ and TN stock data for the upper 25 cm sediment layers in the bays, where the OM pool is partly transient and the ongoing turnover results in a loss of OM during the sediment diagenesis. The burial share of this active layer depends on both the quality of the OM, which differs among OM sources^32,33^, and the microbial communities in the sediment. The latter depends strongly on environmental conditions, such as oxygen levels and sediment disturbance^34^, which are likely to vary along the environmental gradients in the investigated bays. To fully assess the function of the bays as gross and net OM sinks, we would need to also measure C and N pools in deeper sediment layers, as well as the total thickness of sediments with C_org_ and TN content (which may potentially be meter-thick). Shallow bays in Baltic Sea coastal areas have evolved from open to enclosed bay systems due to isostatic land uplift and sedimentation^24^. Roughly, the more enclosed bays are in a later successional stage and therefore have on average accumulated organic matter over a longer time period (centuries), compared to more open bays. This likely results in a larger volume of sediment and potentially larger total stocks, even if the stocks in the upper 25 cm layers were equal or lower in the most enclosed bays.

Compared to the clear effect of bay openness on sediment C_org_ and TN content, the influence of submerged vegetation was less pronounced. The positive correlation between SAV cover and % C_org_ and TN content, respectively, as well as the negative correlation between bare sediment and TN content, may indicate higher organic matter deposition and lower erosion in sites and bays with high vegetation cover. This is in line with the well documented function of submerged vegetation (both seagrasses and freshwater species) in reducing wave energy and current velocity, which promotes sedimentation and decreases resuspension and erosion within SAV meadows^20^. This function has also been documented in semi-enclosed bays of the Baltic Sea during summer, when the vegetation is fully developed^35^. However, the correlation with C_org_ and TN content did not translate into increased sedimentary stocks in the top 25 cm layers and neither % content nor stocks differed between vegetated and non-vegetated patches. This contrasts with the seagrass literature, where a number of studies have shown that C_org_ stocks are often larger within seagrass meadows compared to adjacent areas without seagrass plants^36,37^.

One possible explanation for the lack of correlation between vegetation cover and carbon and nutrient stocks in our study is that the function of vegetation to promote accumulation is overshadowed by the strong influence of openness on hydrodynamic conditions and that vegetation has little impact on sediment dynamics in the enclosed bays with very low hydrodynamic impact. Similarly, a number of studies from seagrass habitats across regions with different hydrographic conditions have failed to observe any effect of seagrass density or biomass on carbon stocks^21,22,31^. This was also proposed as the reason for the lack of relationship between cover or biomass of SAV and C_org_ stocks in estuarine brackish water vegetation in the Mississippi river delta^11^. The patchy structure of the vegetation in several of the investigated bays may also have influenced the relationship, since sediment accumulation can differ widely between large vegetation patches and more scattered vegetation^38^. However, we cannot exclude that the mixed plant communities typical for brackish-water ecosystems may be less consistent than seagrass communities in their function as carbon and nutrient sinks. Notably, the vegetation in the present study consists of a mix of annual and perennial species. *Najas marina*, which dominated the vegetation in a number of the bays is an annual species that is only present in the habitat from early summer to late autumn, while many of the other species dominating in the studied bays (e.g. *Chara tomentosa*, *Stuckenia pectinata* and *Myriophyllum spicatum*) are perennial and keep a certain amount of above-ground biomass also during the winter. This means that the maximum biomass and cover of vegetation, observed when the vegetation is fully developed in late summer or early autumn and used as predictor variables in the PLS analysis, only represents the vegetation characteristics during part of the year. Annual species are also likely to shift their distribution between years, which could explain the lack of significant differences between vegetated and unvegetated patches within bays. Given the varying life-histories of species in these brackish-water communities, it is likely that the importance of SAV for the function as carbon and nutrient sinks varies throughout the year and between bays. Still, the significant effect of vegetation cover on % C_org_ and TN content suggests that the overall vegetation cover in the bays may affect the depositional environment in the bays and that the loss of mixed brackish-water vegetation seen in many Baltic coastal bays^39^, as well as in other estuarine and coastal systems^13,40^, could increase sediment resuspension and erosion of organic matter, potentially reducing the long-term storage of carbon and nutrients in the sediment.

The area of the local catchment had no influence on either content (%) or stocks of C_org_ or TN, but the area of open land in the catchment was positively correlated to sediment C_org_ and TN content. This may be a sign that the characteristics of the local catchment affect the input of organic matter, as well as nutrients that can fuel local production, to the bay. Such an influence of landscape configuration and geomorphic settings on carbon and nutrient storage levels in shallow nearshore systems has been observed in various land-sea settings, where the strength of the sink function may (at least partly) depend on the spatial arrangement of coastal vegetation^18^, geomorphic settings^41^, contemporary land use^42^ and strength of the land-to-sea gradient^43^. In addition, as the configuration and composition of a seascape affects movement and lateral transfer of organic carbon and nutrients^44^, human influences in terms of land-use change, deforestation and urban development may also strongly impact the sedimentary sink function of shallow coastal systems^45,46^. It seems hence obvious that the spatial arrangement of the land-sea continuum plays an important role and should be carefully considered together with determinants at local scale when assessing carbon and nutrient sink capacity of a coastal system^18,36,45^.

The mean C_org_ and TN stocks reported here (2400–4600 g C_org_ m^− 2^ and 270–570 g TN m^− 2^ in the top 25 cm) are in the same range as those reported for seagrass habitats in sheltered and depositional coastal environments^21,22,47^, which are pointed out as hotspots for blue carbon sequestration^14^. Even higher C_org_ stocks (2300–3200 g C_org_ m^− 2^ in the top 10 cm and 10000–19700 in the top 50 cm) were found in comparable brackish water vegetation in the interior parts of the Mississippi river delta, protected behind barrier islands^11^. We did not measure accumulation rates, but previous studies from similar semi-enclosed bays found C_org_ and TN accumulation rates comparable to those in sheltered seagrass habitats (13–50 g C_org_ m^− 2^ yr^− 1^ and 1.3–5 g N_tot_ m^− 2^ yr^− 1^)^48^. Therefore, our study further supports the function of shallow coastal habitats with brackish-water vegetation as carbon and nutrient sinks, which should be considered in regional estimates of these functions in coastal areas. From a coastal management perspective, the results show that the ongoing disturbance of shallow enclosed bays in the Baltic Sea^49,50^, including extensive dredging of shallow areas^51^, risks to disturb and mobilise significant sediment carbon and nutrient stocks, in particular in the most enclosed bays.

In conclusion, we show that shallow, semi-enclosed bays in the Baltic Sea act as reservoirs for carbon and nitrogen, contributing to climate change mitigation and protecting local water quality. The major part of the accumulated carbon originated from land or from pelagic production, which accumulated in higher concentrations in the most enclosed bays. The extent of open land in the catchment area and the cover of submerged vegetation were also significantly correlated with sediment carbon and nitrogen concentrations. However, further studies are needed to determine the importance of landscape configuration and vegetation for carbon and nutrient sequestration in coastal bays. More broadly, the results suggest that morphometrically isolated water-basins in low-saline coastal areas, such as lagoons, estuaries, river mouths and inland seas, constitute significant sinks for carbon and nitrogen and can be considered as blue-carbon habitats.

## Methods

### Study area

The Baltic Sea is a large, enclosed inland sea, where the slow water-exchange with the open ocean and large freshwater inflow from over 200 rivers creates stable brackish-water conditions with smaller salinity fluctuations than in typical estuaries. The study was carried out in the Stockholm archipelago on the Swedish Baltic Sea coast, which includes extensive shallow-water areas and a large number of coastal bays with different degrees of isolation from the sea. We sampled in nine shallow, semi-enclosed bays, selected to represent a gradient from very enclosed to more open bay systems (Fig. 2). Four of the bays are situated in the inner Stockholm archipelago close to the major freshwater outflow from the large lake Mälaren, resulting in a low and variable salinity between 1 and 5 (occasionally < 1 during snowmelt). The remaining five bays are situated in the inner archipelago north of Stockholm, with less influence of large freshwater outflows, and with less variable and slightly higher salinity (between 5.5 and 6). The coast is affected by shoreline displacement due to post-glacial land-uplift, which is currently 5–6 mm per year in the study area^52^.

### Field sampling

Field sampling took place over a three-week period in September 2021. We sampled four stations in each bay, with the exception of one bay (Eke fjärd) where we could only sample three stations due to equipment failure. We used a stratified random sampling scheme, with two stations in the depth interval 0.5 to 1.5 m and the other two between 1.5 and 3 m depth. This captured the depth range occurring in all bays, but excluded the deeper areas of the few bays with larger maximum depth than 3 m. The minimum distance between two sampling points was 40 m.

At each station, we took one sediment core using a Willner gravity corer (length = 50 cm, diameter = 64 mm). The length of the core was measured and a compression factor was calculated following Skilbeck et al.^53^. The average core length was 34.9 cm (SD ± 7.6) and the average compression was 8.3% (± 7.7). Subsequently, the core was cut in three parts: the top sediment (0–5 cm depth), an intermediate sections (5–12.5 cm) and the bottom section (the rest of the core), adjusted for the calculated sediment compression. After sediment sampling, we characterised the benthic vegetation community at the station through snorkelling. The total cover of benthic vegetation as well as the cover of each occurring vegetation species were assessed in a 1 m^2^ quadrat placed around or within 20 cm from the sediment sampling point. At stations with vegetation, we randomly collected a quantitative biomass sample from a 0.04 m^2^ area within the quadrat. Sediment and vegetation samples were kept dark and cool during transport to the laboratory, where they were frozen in -18° C until subsequent analyses. The water depth at the stations was measured with a lead line to the nearest decimeter and corrected for the seawater level at the time of sampling, which was measured at the Swedish Meteorological and Hydrological Institute’s (SMHI’s) closest sea level measurement station (located in Stockholm).

In three of the studied bays (Fårholmsfladen, Handfatet and Söderfladen), we did an additional sampling to assess the small-scale variation between patches with bare substrate and patches with dense submerged vegetation dominated by either the charophyte *Chara tomentosa* (in Söderfladen) or the vascular plant *Najas marina* (in Fårholmsfladen and Handfatet). This sampling was done in one restricted, 500–1000 m^2^, area in each bay. The sampling areas were chosen in the field using two main criteria. The area should be: (1) a mosaic of vegetation patches (at sizes of more than ten square metres) intermixed with areas without vegetation, and (2) a flat or gently sloping seabed with a maximum depth range of 0.3 m in the area. The water depth (corrected for seawater level) was 0.5–0.8 m, 0.5–0.7 m and 0.4–0.7 m in the three sampling areas, which means that the sampling represents the conditions in the shallowest parts of the bays. We collected eight sediment cores in each of the three sampling areas, four in vegetated patches and four in bare patches, using a syringe corer (length = 10 cm, diameter = 2.7 cm) operated by a snorkeler. The samples were taken in the middle of different vegetated or bare patches, with a distance of at least 1 m to the patch edge, ensuring that the samples from both patch types were equally dispersed over the sampling area and with a minimum distance of 10 m between two samples. Subsequent to the sediment sampling, we took a quantitative sample of the vegetation from a 0.04 m^2^ area around the sampling point. The top sediment (0–5 cm depth) was taken from each core and the sediment and vegetation samples were handled in the same way as the main sampling procedure in all nine bays.

### Sample processing

After defrosting, sediment samples were homogenised and divided into two subsamples; one for analysis of sediment weight, carbon and nitrogen content as well as stable isotopes, and the other for grain-size analysis. All subsamples were cleaned of roots, shells and benthic fauna and dried at 60° C to constant weight. The subsamples for element analyses were first weighted to determine dry weight and then ground using a mortar and pestle or a mixing mill and analysed for organic carbon (C_org_) and total nitrogen (TN) content and stable isotopes using a mass spectrometer (CN elemental analyzer, Flash EA1112) connected to an isotope ratio mass spectrometer (Delta V2, Thermo Fisher Scientific). Samples for analysis of organic carbon were pre-treated with 1 M HCl to eliminate carbonates. The subsamples for grain size analysis were treated with 10% H_2_O_2_ until the reaction ceased, to remove organic matter. Grain size was analysed through wet sieving with mesh sizes 2, 1, 0.5, 0.2, 0.125 and 0.063 mm and drying the fractions at 60 °C to determine dry mass. The sediment mud content is given as dry mass percent of the fraction < 0.063 mm.

The quantitative vegetation samples were defrosted and rinsed with water to remove sediment. All macroscopic plants and macroalgae were identified to species and the aboveground parts were separated and dried in 60 °C for 48 h to determine the dry weight.

### Landscape and vegetation predictors

As a measure of bay isolation, we used topographic openness (Ea), calculated according to Håkansson^26^ and Persson et al.^25^ using Eq. (1), where A_t_ is the narrowest cross-section of the bay opening(s) (calculated from depth and width) and a is the water surface area of the bay.

1 $${\text{Ea}}\,=\,{\text{1}}00 \times \left( {{{\text{A}}_{\text{t}}}/{\text{ a}}} \right)$$

Depth was measured with a lead line in the field, with measurements every 10 m across the opening, and adjusted to mean sea water level. The width of the opening and water surface area were measured from georeferenced aerial photographs (Google Earth, https://www.google.com/earth or orthophotos from “Digitala kartbiblioteket” SLU - https://maps.slu.se/get*)* using GIS.

Catchment characteristics were derived by first delineating the bays’ local catchments in GIS, using maps of hydrography (*“Hydrography Download”*) and detailed topography (*“Elevation model Download”*) from the Swedish Land Survey (Lantmäteriet). Subsequently, the catchment maps were overlaid with land cover data from the national Swedish cadastral map (*“Land cover Download”*) to measure the area of different land cover (forest/open land/agricultural land/water) in the catchments. It is important to note that these variables describe the local catchments, while the bays also receive water and organic matter from the archipelago area outside the bay. The latter may also affect carbon and nutrient accumulation in the bays, but this could not be tested here because many of the bays were situated in the same larger archipelago area (Fig. 2).

Vegetation characteristics were derived both for the vegetation community directly around the sampling stations (from the vegetation survey during sediment sampling) and for the vegetation community in the entire bay. The latter was based on vegetation surveys in August and early September from at least one of the years between 2019 and 2021, which were available for eight of the studied bays. The surveys involved underwater inventory conducted through snorkelling within four to nine circular plots per year, each with a radius of 5 m (equating to approximately 80 square metres). The plots were spread evenly from the entrance to the innermost part of the bays, with random allocation of the plots within subareas along a distance-from-entrance gradient, excluding nearshore areas with less than 0.5 m water depth.

For both datasets (local and bay-level surveys), we calculated (a) cumulative vegetation cover (SAVCov) as the sum of the cover of all recorded species except filamentous algae, (b) cover of unvegetated bottom (UnvegCov), and (c) cover of filamentous algae (AlgCov). For the local dataset, we also calculated total above-ground vegetation biomass (SAVBiomass). All variables were calculated as mean values from all study plots and for the three bays with vegetation surveys from two or three years, we calculated mean values from all years.

In addition to the submerged vegetation characteristics described above, we also measured the percent cover of emergent reed along the shores of the bay (ReedCov). The reed belt extent was manually delimited using QGIS (v3.34.3) from georeferenced aerial images from late spring or early summer, when reed belts are fully developed. The aerial images were orthophotos with a 0.25 m resolution from the years 2009, 2013, 2015 and 2017, downloaded from “Digitala kartbiblioteket” (SLU - https://maps.slu.se/get*).* The reed extends over the water line and to derive the part of the reed belt below the water line, the polygons of reed extent were intersected with the mean water level. The reed cover in the bay was calculated as the mean for two to four years, depending on the years available for each bay.

### Data analyses

The inorganic carbon was subtracted from the total organic carbon for each sample, and then the organic carbon per unit area (g C_org_ per cm^2^) was calculated for each section of the cores. The inorganic carbon content was low (0.05–6.37% of the total carbon in the sediment), which is typical for Baltic Sea sediments with low levels of carbonates^30^.

Sediment density (i.e. dry bulk density, DBD, in g per cm^3^) was calculated with Eq. (2), where M_s_ is the dry mass of the sample (in g) and V_s_ is the volume of the wet sample (in cm^3^), following Smeaton et al.^53^.

2 $${\text{DBD}}\,=\,{{\text{M}}_{\text{s}}}/{{\text{V}}_{\text{s}}}$$

One-way ANOVAs were used to evaluate if C_org_ and TN content (in % of dry mass) and stocks in the upper 25 cm of sediment differed between the nine bays, using R (version 3.2.1)^54^. Visual inspection of histograms of the raw data and residual plots were used to identify deviations from homoscedasticity and normality and to identify potentially influential outliers (here and in subsequent analyses). For % N content, the data deviated from homoscedasticity and the analysis of this variable was done with GLS (generalized least squares) using the *nlme* package^55,56^. We allowed for different variances for the different bays, which resulted in homogeneous residual variance and a better model according to AIC (decreased from 22.9 to 9.0) and log likelihood ratio test (log ratio = 29.9, *p* < 0.001).

We used partial least squares (PLS) regression modelling to assess the relative importance of selected predictor metrics including ten landscape scale variables, four local-scale vegetation or habitat variables and two sediment variables (see caption of Fig. 4) on levels of C_org_ and TN content in % of dry mass content and in stocks per area. This was performed by modelling projections to latent structures, that is, variables with the best predictive power^57^. All PLS analyses were carried out on untransformed data using the SIMCA 17 software (UMETRICS, Malmö, Sweden). PLS modelling technique is useful when the data include a large number of predictors and one has to deal with multicollinearity^58^. Various recent studies confirm the utility of this method when working with different types of ecological data^45,59,60^.

The data from the small-scale study in three bays was analysed with two-way ANOVAs in R, testing the effect of patch type (vegetation or no vegetation) and bay on sedimentary content of carbon and nitrogen (both as percentage of dry weight and as weight per area) in the upper 5 cm. Due to the variability in extent and species composition of the SAV habitats in shallow Baltic Sea bays, we did not expect a strong relationship with sediment characters in the deeper parts of the core. Percentage content of both C_org_ and TN showed strong heteroscedasticity, and the variation between samples differed strongly between bays. Rank transformation resulted in homogenous variance of the residuals; therefore, we performed the analysis on transformed data. The dry weight of carbon and nitrogen per area met the assumptions of homoscedasticity and normality, but both had one problematic outlier (the same data point for both variables). We therefore ran the analysis both with and without this data point and report the results from both models.

## Electronic supplementary material

Below is the link to the electronic supplementary material.

Supplementary Material 1

## Data Availability

The datasets used in the current study is published on Figshare (10.6084/m9.figshare.28228952).
