# Supplementary material for: Influence of landscape characteristics and submerged aquatic vegetation on sediment carbon and nitrogen storage in shallow brackish water habitats
Source: Sci Rep. 2025 Mar 6;15:7808. doi: 10.1038/s41598-025-92217-z (PMC11885652; doi:10.1038/s41598-025-92217-z)
Supplement: Supplementary file 1 — Supplementary Material 1 [file 41598_2025_92217_MOESM1_ESM.pdf]

Appendix to submitted manuscript: Influence of landscape characteristics on sediment carbon and nitrogen storage in brackish vegetation habitats

Sofia A. Wikström, Betty Gubri, Maria E. Asplund, Martin Dahl, Martin Gullström, Joakim P. Hansen, Linda Kumblad, Emil Rydin, Andrius Garbaras, Mats Björk

Supplementary Figure 1

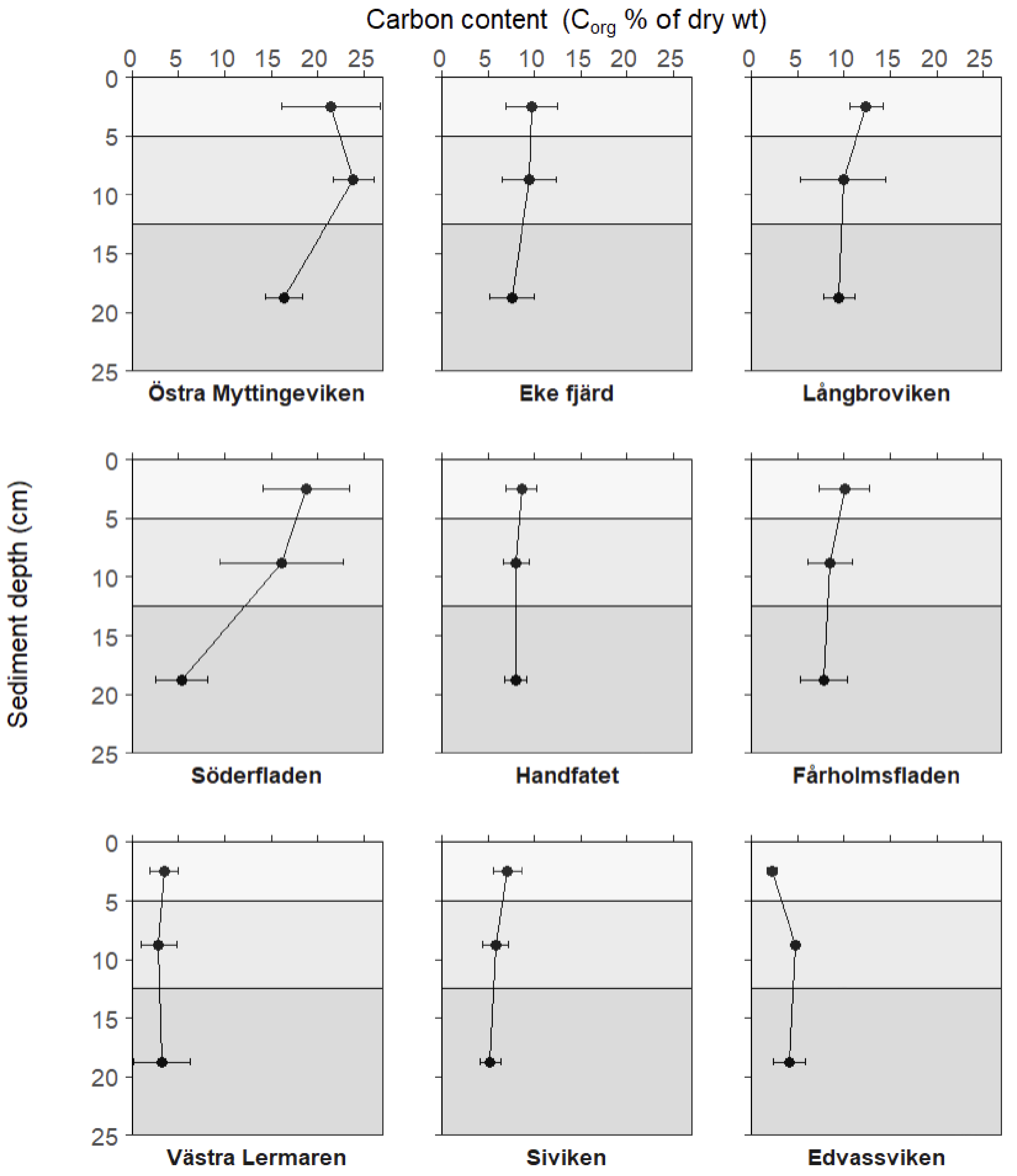

Depth profiles of organic carbon content in the nine bays, ordered from smallest to largest bay topographic openness (Ea). Data are means and standard deviation for 3 – 4 cores in each bay, divided in three sections (0 – 5, 5 – 12.5 and 12.5 – 25 cm from the sediment surface).

Supplementary Figure 2

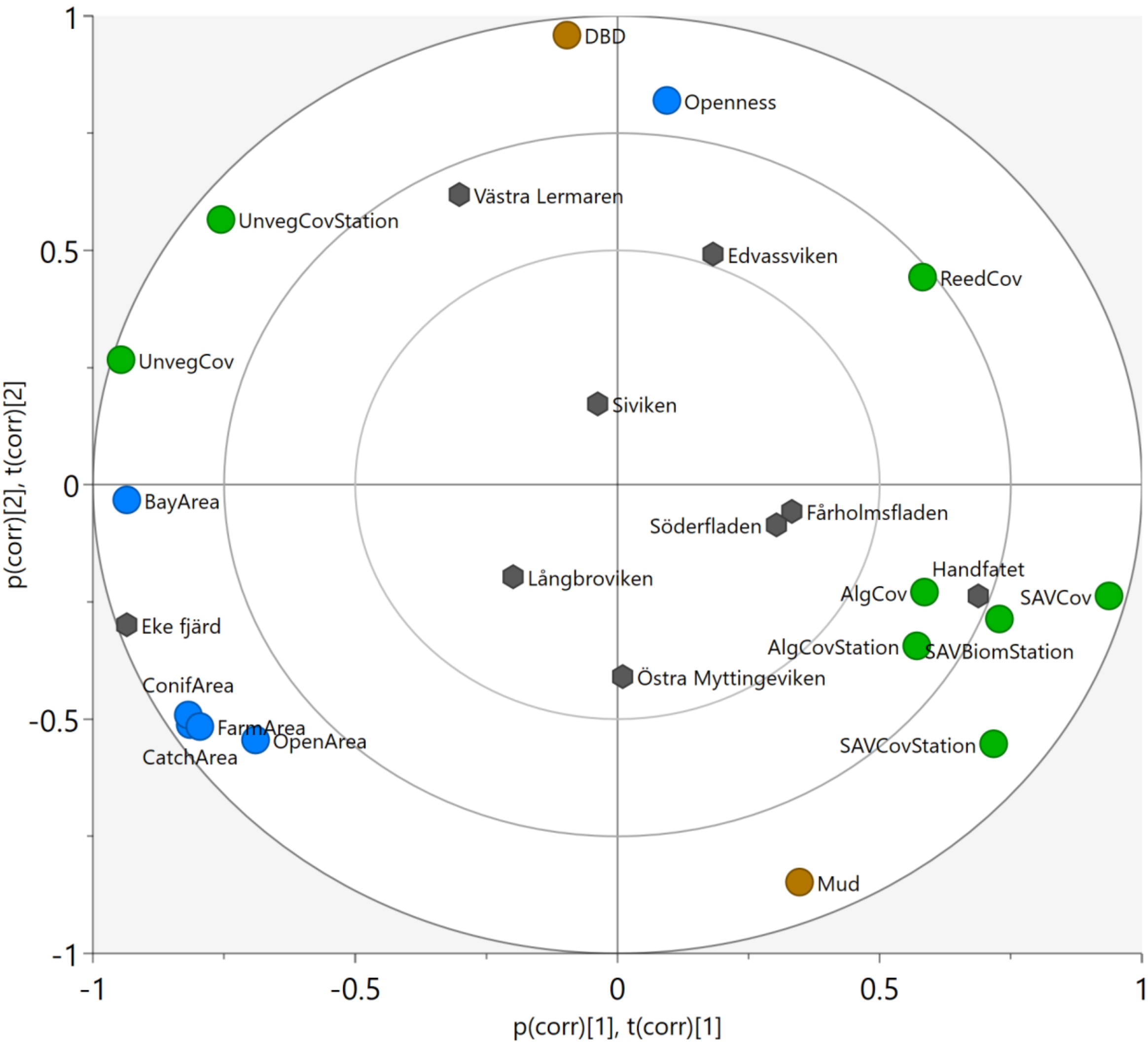

Principal component analysis (PCA) biplot that visually summarises the relationship between the 9 sites and landscape variables (blue), the sediment properties (brown) and submerged vegetation variables or unvegetated areas (green). The biplot-model covers in total 77% (R2X(cum) of the correlative variation in the data, whereof Comp 1 (x-axis) 49% and Comp 2 (y-axis) 29%. Explanation for variable abbreviations: Openness = Bay topographic openness (Ea), BayArea = Area of bay, CatchArea = Catchment area, ConifArea = Area of coniferous forest in catchment, OpenArea = Area of open land in catchment, FarmArea = Area of farmland in catchment, SAVCov = Cover of submerged vegetation, ReedCov = Cover of aquatic reed, AlgCov = Cover of filamentous algae, UnvegCov = Cover of bare sediment, SAVCovStation = Cover of submerged vegetation at sampling stations, AlgCovStation = Cover of filamentous algae at sampling stations, UnvegCovStation = Cover of bare sediment at sampling stations, SAVBiomStation = Above-ground biomass of submerged vegetation at sampling stations, DBD = Sediment dry bulk density, Mud = Mud content in sediment. See Methods for more details on the predictor variables.

Supplementary Figure 3

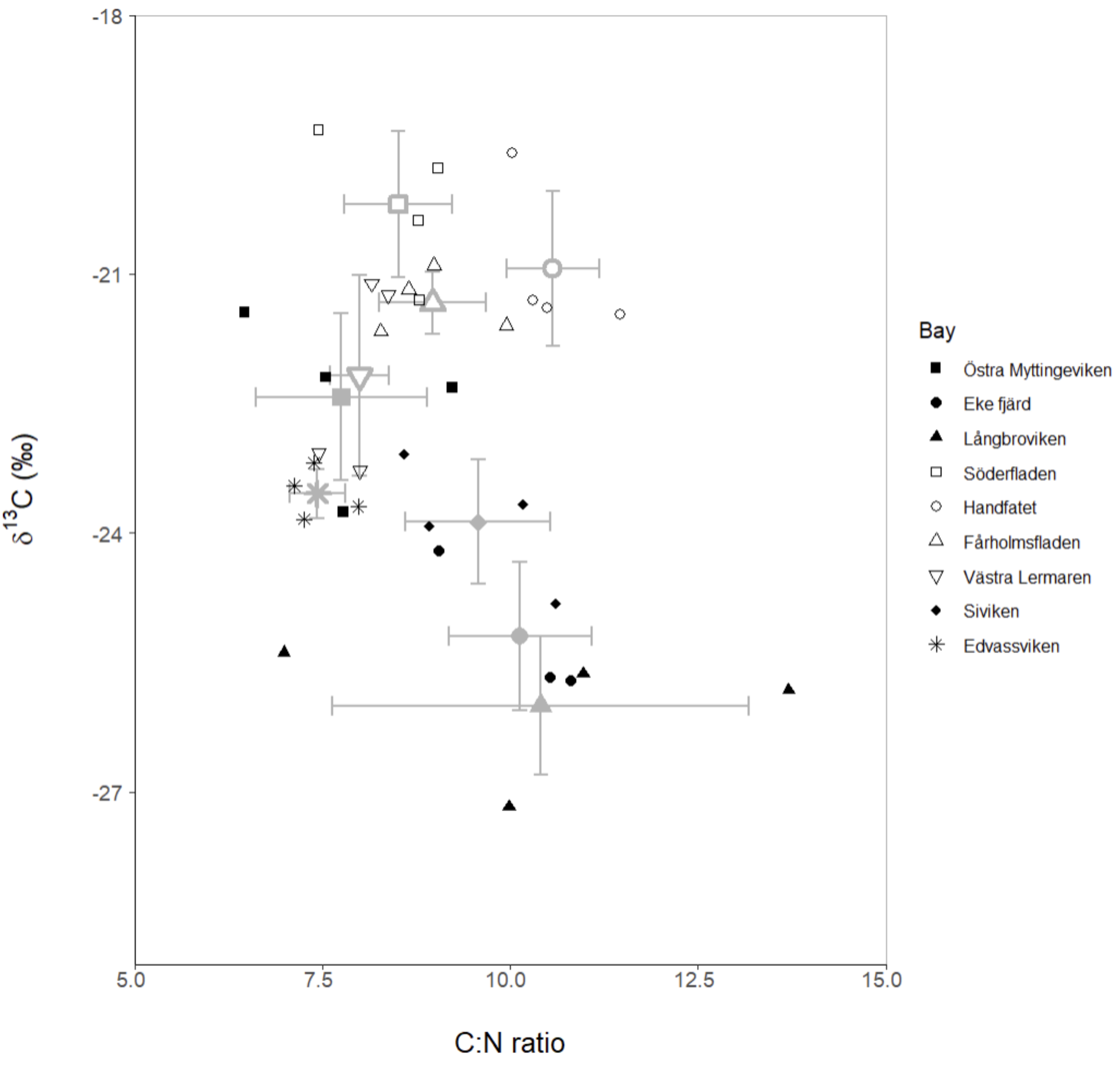

Sedimentary $\delta^{13}C$ in the upper 25 cm in the nine bays. The plot shows mean values for the cores (in black) and mean and SD for the bays (in grey).
